# Higher-order numerical scheme for linear quadratic problems with bang–bang controls

**DOI:** 10.1007/s10589-017-9948-z

**Published:** 2017-10-06

**Authors:** T. Scarinci, V. M. Veliov

**Affiliations:** 1 Department of Statistic and Operation Research, University of Vienna, Vienna, Austria; 2 Institute of Statistics and Mathematical Methods in Economics, Vienna University of Technology, Vienna, Austria

**Keywords:** Optimal control, Numerical methods, Bang–bang control, Linear-quadratic optimal control problems, Time-discretization methods, 49M25, 65L99, 49J30, 49N10, 49J15

## Abstract

This paper considers a linear-quadratic optimal control problem where the control function appears linearly and takes values in a hypercube. It is assumed that the optimal controls are of purely bang–bang type and that the switching function, associated with the problem, exhibits a suitable growth around its zeros. The authors introduce a scheme for the discretization of the problem that doubles the rate of convergence of the Euler’s scheme. The proof of the accuracy estimate employs some recently obtained results concerning the stability of the optimal solutions with respect to disturbances.

## Introduction

Discretization schemes for optimal control problems have been largely investigated in the last 60 years (see, e.g., [6–9, 17, 21], and the more recent paper [5] and the references therein). In the aforementioned papers and in most of the literature, the optimal controls are typically assumed to be sufficiently smooth (at least Lipschitz continuous) and results are usually based on second-order optimality conditions. On the other hand, whenever the control appears linearly in the system, the lack of coercivity typically leads to discontinuities of the optimal controls.

Recently, new second-order optimality conditions for systems that are linear with respect to the control have been developed. We refer to [3, 22] for analysis of second-order necessary conditions for bang–bang and singular-bang controls, respectively. Results on the stability of solutions with respect to disturbances were also recently obtained, see [4, 12, 14, 25] and the bibliography therein. Based on these results, error estimates for the accuracy of the Euler discretization scheme applied to various classes of affine optimal control problems were obtained in [1, 2, 13, 18, 26, 27]. The error estimates are at most of first order with respect to the discretization step, which is natural in view of the discontinuity of the optimal control. For the same reason, using higher order Runge–Kutta discretization schemes on a fixed grid does not help to improve the order of accuracy. Seemingly, the first paper that addresses the issue of accuracy of discrete approximations for affine problems is [30], where a higher order Runge–Kutta scheme is applied to a linear system, but the error estimate is of first order or less.

A new type of discretization scheme was recently presented in [23] for Mayer’s problems for linear systems. The idea behind this scheme goes back to [15, 20, 28] and is based on a truncated Volterra–Fliess-type expansion of the state and adjoint equations. The analysis of the convergence and of the error estimate makes use of the *strong Hölder metric sub-regularity* of the map associated with the Pontryagin maximum principle, proved in [25].

The goal of the present paper is to extend this discretization scheme and the pertaining error analysis to affine linear-quadratic problems. This extension is not a routine task, due to the appearance of the state function in the associated with the problem switching function, and of both the state and the control, in the adjoint equation.

More precisely, we consider the problem

1.1 $$\begin{aligned} J(x,u)&:= \frac{1}{2} x(T)^{\top } Q x(T) + q^{\top } x(T)\nonumber \\&\quad \;+ \int _0^T \left( \frac{1}{2} x(t)^{\top }W(t)x(t) +x(t)^{\top }S(t)u(t) \right) {\mathrm{\,d}}t \longrightarrow \min \end{aligned}$$

subject to

1.2 $$\begin{aligned} \dot{x}(t)= & {} A(t)x(t)+B(t)u(t), \quad x(0)=x_0, \qquad \text{ for } \text{ a.e. } t\in [0,T], \end{aligned}$$

1.3 $$\begin{aligned}&u(t)\in U:=[-1,1]^m \qquad \text{ for } \text{ a.e. } t \in [0,T]. \end{aligned}$$

Here [0, *T*] is a fixed time horizon, the state *x* is *n*-dimensional, the initial state $x_0$ is given, the control *u* is *m*-dimensional, $Q\in {\mathbb {R}}^{n\times n}, q\in {\mathbb {R}}^n$, and the matrix functions $A,\, W:[0,T]\rightarrow {\mathbb {R}}^{n\times n}$ and $S,\, B:[0,T]\rightarrow {\mathbb {R}}^{n\times m}$ are given data; the superscript “${}^\top$” denotes transposition. Admissible controls are all measurable functions with values in the set *U* for a.e. $t \in [0,T]$. Linear terms are not included in the integrand in (1.1), since they can be shifted in a standard way into the differential equation (1.2).

The optimal controls in the problem (1.1)–(1.3) are typically concatenations of bang–bang and singular paths. In this paper, we assume that the optimal controls are of strictly bang–bang type with a finite number of switches, and the components of the switching function have a certain growth rate at their zeros, characterized by a number $\kappa \ge 1$. This number appears in the error estimate obtained in this paper for the proposed discretization scheme. “Generically”, $\kappa = 1$, and in this case the error estimate is of second order.

The paper is organized as follows. In Sect. 2 we recall some notations and formulate the assumptions. In Sect. 3 we introduce our discretization scheme and present the main result—the error estimate. Section 4 contains the proof. Section 5 presents an error estimate in case of inexact solution of the discretized problem. A numerical experiment confirming the theoretical findings is given in Sect. 6. Concluding remarks and further perspectives are discussed in Sect. 7.

## Preliminaries

First of all we pose some assumptions, which are standard in the context of problem (1.1)–(1.3).

### Assumption (A1)

The matrix functions *A*(*t*), *B*(*t*), *W*(*t*) and *S*(*t*), $t \in [0,T]$, have Lipschitz continuous first derivatives, *Q* and *W*(*t*) are symmetric.

The set of all admissible controls will be denoted by ${\mathcal {U}}\subset L^\infty$. A pair (*x*, *u*) formed by an admissible control *u* and the corresponding solution *x* of (1.2) is referred to as an admissible process, and the set of all admissible processes is denoted by ${\mathcal {F}}$. The set ${\mathcal {F}}$ will be considered as a subset of the space $W^{1,1}_{x_0} \times L^{1}$, where $W^{1,1}_{x_0} = W^{1,1}_{x_0}(0,T)$ is the (affine) space of all absolutely continuous functions $x:[0,T]\rightarrow {\mathbb {R}}^n$ with $x(0) = x_0$, and $L^{1}= L^{1}(0,T)$ has the usual meaning.^1^

Due to the compactness and the convexity of *U*, the set ${\mathcal {F}}$ is compact with respect to the $L^2$-weak topology for *u* and the uniform norm for *x*. Thus a minimizer $(\hat{x},\hat{u})$ does exist in the space $W^{1,1}_{x_0}\times L^{1}$ (in fact, also in $W^{1,\infty }_{x_0} \times L^{\infty }$). The following assumption requires a sort of “directional convexity” of the objective functional *J* at $(\hat{x}, \hat{u})$.

### Assumption (A2)

$$\begin{aligned}&\frac{1}{2}z(T)^{\top }Qz(T) + \int _0^T \left( \frac{1}{2}z(t)^{\top } W(t) z(t) + z(t)^{\top }S(t)v(t) \right) {\mathrm{\,d}}t \ge 0\\&\quad \text{ for } \text{ every } (z,v) \in \mathcal {F} - (\hat{x}, \hat{u}). \end{aligned}$$

Let $(\hat{x},\hat{u})$ be an optimal process in problem (1.1)–(1.3). According to the Pontryagin (minimum) principle, there exists $\hat{p}\in W^{1,\infty }$ such that $(\hat{x},\hat{u},\hat{p})$ satisfies the following system of generalized equations: for a.e. $t\in [0,T]$,

2.1 $$\begin{aligned}&0= \dot{x}(t)-A(t)x(t)-B(t)u(t), \;\; x(0)=x_0, \end{aligned}$$

2.2 $$\begin{aligned}&0= \dot{p}(t)+A(t)^{\top }p(t)+W(t)x(t)+S(t)u(t), \end{aligned}$$

2.3 $$\begin{aligned}&0\in B(t)^{\top }p(t)+S(t)^{\top } x(t)+N_U(u(t)), \end{aligned}$$

2.4 $$\begin{aligned}&0=p(T)-Q x(T) -q, \end{aligned}$$

where $N_U(u)$ is the normal cone to *U* at *u*:

$$\begin{aligned} N_U(u) = \left\{ \begin{array}{cl} \emptyset &{} \quad \text {if }u \not \in U,\\ \{ l \in {\mathbb {R}}^m : \,\langle l, v-u \rangle \le 0 \; \forall v \in U \} &{} \quad \text {if }u \in U. \end{array} \right. \end{aligned}$$

System (2.1)–(2.4) can be shortly rewritten as

2.5 $$\begin{aligned} 0 \in F(x,p,u), \end{aligned}$$

where *F* is the set-valued map defined by

$$\begin{aligned} F(x,p,u):= \left( \begin{array}{c} \dot{x} - A x - B u \\ \dot{p} + A ^{\top }p + W x +S u \\ B^{\top }p+S^{\top } x +N_{\mathcal {U}}(u) \\ p(T)-Q x(T) -q \end{array} \right) . \end{aligned}$$

The mapping *F* is considered as acting from the space ${\mathcal {X}}$ to the space ${\mathcal {Y}}$, where

$$\begin{aligned} {\mathcal {X}}:= W^{1,1}_{x_0} \times W^{1,1} \times L^1, \qquad {\mathcal {Y}}:= L^1 \times L^{1} \times L^\infty \times {\mathbb {R}}^n. \end{aligned}$$

The norms in these spaces are defined as usual: for $(x,p,u) \in {\mathcal {X}}$ and $(\xi ,\pi ,\rho ,\nu ) \in {\mathcal {Y}}$,

$$\begin{aligned} \Vert x \Vert _{1,1} + \Vert p \Vert _{1,1} + \Vert u \Vert _1 \end{aligned}$$

and

$$\begin{aligned} \Vert (\xi ,\pi ,\rho ,\nu ) \Vert := \Vert \xi \Vert _{1}+\Vert \pi \Vert _{1} + \Vert \rho \Vert _{\infty } + | \nu |, \end{aligned}$$

where $\Vert x\Vert _{1,1}$ abbreviates $\Vert x\Vert _{W^{1,1}}$ and $\Vert \cdot \Vert _{s}$ is the norm in $L^s$, $s \in \{1, \infty \}$. Notice that the normal cone $N_{\mathcal {U}}(u)$ to the set ${\mathcal {U}}\subset L^1$ at $u(\cdot ) \in {\mathcal {U}}$ has the point-wise representation $\lbrace \xi \in L^\infty : \xi (t) \in N_U(u(t)) \text{ a.e. } \text{ on } [0,T]\rbrace$.

We also define the distance

$$\begin{aligned} d^{\#} (u_1,u_2)= \text{ meas } \left\{ t\in [0,T]: u_1(t)\ne u_2(t) \right\} \end{aligned}$$

in the space $L^\infty$. We mention that ${\mathcal {U}}\subset L^\infty$ is a complete metric space with respect to this metric ([11, Lemma 7.2]).

Observe that the inclusion (2.3) is equivalent to $u(t)\in \mathop {\text{ Argmin }}\limits _{w\in U} \sigma (t)^{\top }w$, where $\sigma :[0,T]\rightarrow {\mathbb {R}}^m$ is the so-called *switching function*, defined for all $t \in [0,T]$ as

$$\begin{aligned} \sigma (t) = B(t)^{\top }\hat{p}(t)+S(t)^{\top } \hat{x}(t). \end{aligned}$$

Thus, for $$j=1,\ldots , m$$,

2.6 $$\begin{aligned} \hat{u}^j(t) = \left\{ \begin{array}{cl} -1 &{}\quad \text{ if } \sigma ^j(t)>0,\\ 1 &{} \quad \text{ if } \sigma ^j(t)<0,\\ \end{array} \right. \end{aligned}$$

where $\sigma ^j$ and $\hat{u}^j$ stay for the *j*-component of $\sigma$ and $\hat{u}$, respectively.

### Assumption (B)

(*Strict bang–bang property*) There exist positive real numbers $\kappa \ge 1$, $m_0$ and $\delta$ such that for every $j=1,\ldots ,m$, and for every zero $\tau \in [0,T]$ of $\sigma ^j$, the inequality $|\sigma ^j (t)| \ge m_0 | t-\tau |^{\kappa }$ holds for all $t\in [\tau -\delta ,\tau +\delta ] \cap [0,T]$.

### Remark 2.1

Clearly, Assumption (B) implies that each component $\sigma ^j$ has a finite number of zeros in [0, *T*] and then each component of $\hat{u}$ is piecewise constant with values $-1$ and 1.

The following theorem plays a crucial role in the error analysis of the discretization scheme presented below. It is a modification (under weaker conditions and a different space setting) of [4, Theorem 8], and is proved in [24].

### Theorem 2.2

Let Assumption (A1) and (A2) be fulfilled. Let $(\hat{x},\hat{p},\hat{u})$ be a solution of generalized equation (2.5) and let Assumption (B) be fulfilled with some real number $\kappa \ge 1$. Then for any $b > 0$ there exists $c>0$ such that for every $y := (\xi ,\pi ,\rho ,\nu ) \in \mathcal {Y}$ with $\Vert y \Vert \le b$, there exists a triple $(x,p,u)\in \mathcal {X}$ solving $y \in F(x,p,u)$ and every such triple satisfies the inequality

2.7 $$\begin{aligned} \Vert x-\hat{x}\Vert _{1,1}+ \Vert p-\hat{p}\Vert _{1,1} + \Vert u-\hat{u}\Vert _1 \le c \Vert y \Vert ^{1/\kappa }. \end{aligned}$$

We mention that the above property of the mapping *F* and the reference point $(\hat{x},\hat{u},\hat{p}) \in {\mathcal {X}}$ and $0 \in {\mathcal {Y}}$ is a stronger version (non-local with respect to (*x*, *p*, *u*)) of the so-called *metric sub-regularity* [10].

## Discretization scheme

In this section we propose a discretization scheme for problem (1.1)–(1.3) which has a higher accuracy than the Euler scheme without a substantial increase of the numerical complexity of the discretized problem. We recall that the Euler method has already been profoundly investigated in the case where bang–bang controls appear (e.g. [1, 2, 13, 18, 26]). As mentioned in the introduction, in doing this we use an idea that originates from [15, 28] and was implemented in [23] in the case of Mayer’s problems. The approach uses second order truncated Volterra–Fliess series, as described in the next subsection.

### Truncated Volterra–Fliess series

Given a natural number *N*, denote $h = T/N$, and $t_{i}= i h$ for $i=0,\ldots ,N$. Let *u* be an admissible control on $[t_i, t_{i+1}]$. The solution *x* of (1.2) on the interval $[t_i, t_{i+1}]$ can be represented as (see [23, Section 3])

3.1 $$\begin{aligned} x(t)&= \left[ I + (t-t_i) A + \frac{(t-t_i)^2}{2} (A^2 + A') \right] x(t_i) + (B + (t-t_i) AB) \int _{t_i}^t u(s) {\mathrm{\,d}}s\nonumber \\&\quad + (-AB + B') \int _{t_i}^t (s-t_i) u(s) {\mathrm{\,d}}s + O(h^3), \end{aligned}$$

where for shortness we skip the fixed argument $t_i$ in the appearing functions, that is, $A:=A(t_i)$, $B:=B(t_i)$, etc. As usual, we denote by $O(\varepsilon )$, $\varepsilon > 0$, any function that satisfies $|O(\varepsilon )|/\varepsilon \le c$, where *c* is a “generic” constant, that is, depending only on the data of the problem (thus, independent of *i* and *t*, although $O(h^3)$ may depend on *i* and *t* in the above context). The second-order expansion of the solution of the adjoint equation (2.2) differs from that in [23, Section 3] due to the presence of an integral term in the objective functional (1.1), therefore we derive it below. For all $t\in [t_i,t_{i+1}]$,

3.2 $$\begin{aligned} p(t)= \int _{t}^{t_{i+1}}\left( A(s)^{\top } p(s)+W(s)x(s)+ S(s)u(s) \right) {\mathrm{\,d}}s + p(t_{i+1}). \end{aligned}$$

Applying the first order Taylor expansion for *A*, *W*, *S* at $t_i$, the representations (3.1) of *x*(*s*) and (3.2) of *p*(*s*), and skipping all third order terms we obtain that

$$\begin{aligned} p(t)= & {} \int _{t}^{t_{i+1}} \left( A+ (s-t_i)A'\right) ^{\top } \left( p(t_{i+1})\right. \\&\left. + \int _{s}^{t_{i+1}}\left( A^{\top } p(t_{i+1}) +W x(t_i) + S u(\zeta ) \right) {\mathrm{\,d}}\zeta \right) {\mathrm{\,d}}s \\&+\,\int _{t}^{t_{i+1}} \left( W+ (s-t_i) W'\right) \left( x(t_i) + (s-t_i) Ax(t_i) + B \int _{t_i}^s u(\zeta ) {\mathrm{\,d}}\zeta \right) {\mathrm{\,d}}s \\&+\, \int _{t}^{t_{i+1}} \left( S u(s)+(s-t_i)S' u(s) \right) {\mathrm{\,d}}s + p(t_{i+1}) +O(h^3). \end{aligned}$$

Hence, we obtain the following truncated Volterra–Fliess expansion for the adjoint function:

3.3 $$\begin{aligned} \begin{aligned} p(t)&= \left( I+ (t_{i+1}-t)A^{\top } + \frac{ h^2- (t-t_i)^2 }{2} A'^{\top } \right) p(t_{i+1}) \\&\quad + \frac{(t_{i+1}-t)^2}{2} A^{\top } \left( A^{\top }p(t_{i+1}) + W x(t_i) \right) \\&\quad + A^{\top } S \int _{t}^{t_{i+1}} \int _s^{t_{i+1}} u(\zeta ) {\mathrm{\,d}}\zeta {\mathrm{\,d}}s \\&\quad +(t_{i+1}-t) W x(t_i) + \frac{h^2-(t-t_i)^2}{2} (W'+ W A)x(t_i)\\&\quad + WB \int _t^{t_{i+1}}\int _{t_i}^s u(\zeta ) {\mathrm{\,d}}\zeta {\mathrm{\,d}}s \\&\quad + S \int _{t}^{t_{i+1}} u(s) {\mathrm{\,d}}s + S' \int _{t}^{t_{i+1}} (s-t_i) u(s) {\mathrm{\,d}}s + O(h^3). \end{aligned} \end{aligned}$$

Now we shall derive a second order approximation to the integral term of the objective functional *J*(*x*, *u*) on $[t_i,t_{i+1}]$ in term of the first and second order momentum of the controls.

Concerning the quadratic term in the *x*-variable we make use of the first order part of representation (3.1) and of the Taylor expansion at $t_i$ for *W*. Remembering that *W* is a symmetric-matrix-valued function, we obtain that

$$\begin{aligned} \int _{t_i}^{t_{i+1}} x(t)^{\top } W(t) x(t) {\mathrm{\,d}}t= & {} \int _{t_i}^{t_{i+1}}\left( x(t_i) + (t-t_i) A(t_i) x(t_i) + B(t_i) \int _{t_i}^t u(s) {\mathrm{\,d}}s \right) ^{\top }\\&\quad (W(t_i)+(t-t_i)W'(t_i))\left( x(t_i) + (t-t_i) A(t_i)x(t_i) \right. \\&\left. + B(t_i)\int _{t_i}^t u(\tau ) {\mathrm{\,d}}\tau \right) {\mathrm{\,d}}t +O(h^3)\\= & {} h x(t_i)^{\top } \left( W(t_i) + h W(t_i)A(t_i)+ \frac{h}{2} W'(t_i) \right) x(t_i)\\&+ 2 x(t_i)^{\top } W(t_i) B(t_i) \int _{t_i}^{t_{i+1}} \int _{t_i}^t u(s) {\mathrm{\,d}}s {\mathrm{\,d}}t + O(h^3). \end{aligned}$$

Note that an easy calculation implies that

3.4 $$\begin{aligned} \int _{t_i}^{t_{i+1}}\int _{t_i}^t u(\tau ) {\mathrm{\,d}}\tau {\mathrm{\,d}}t = h \int _{t_i}^{t_{i+1}} u(t) {\mathrm{\,d}}t - \int _{t_i}^{t_{i+1}} (t-t_i) u(t) {\mathrm{\,d}}t. \end{aligned}$$

Now we consider the mixed term in the integral in (1.1). A calculation of the same fashion as the previous one yields

3.5 $$\begin{aligned} \int _{t_i}^{t_{i+1}} x(t)^{\top } S(t)u(t) {\mathrm{\,d}}t= & {} \int _{t_i}^{t_{i+1}} \left( x(t_i) + (t-t_i)A(t_i)x(t_i)+ B(t_i)\int _{t_i}^t u(s) {\mathrm{\,d}}s \right) ^{\top } \nonumber \\&\quad \left( S(t_i)+S'(t_i)(t-t_i) \right) u(t) {\mathrm{\,d}}t +O(h^3) \nonumber \\= & {} x(t_i)^{\top }\left( S(t_i)\int _{t_i}^{t_{i+1}} u(t) {\mathrm{\,d}}t + S'(t_i)\int _{t_i}^{t_{i+1}} (t-t_i) u(t) {\mathrm{\,d}}t \right) \nonumber \\&+\, (A(t_i)x(t_i))^{\top } S(t_i) \int _{t_i}^{t_{i+1}} (t-t_i)u(t) {\mathrm{\,d}}t \nonumber \\&+ \int _{t_i}^{t_{i+1}}\left( B(t_i) \int _{t_i}^t u(s) {\mathrm{\,d}}s \right) ^{\top } S(t_i) u(t) {\mathrm{\,d}}t + O(h^3).\nonumber \\ \end{aligned}$$

Let us focus on the last term:

$$\begin{aligned}&\int _{t_i}^{t_{i+1}}\left( B(t_i) \int _{t_i}^t u(s) {\mathrm{\,d}}s\right) ^{\top } S(t_i) u(t) {\mathrm{\,d}}t \\&\quad = \int _{t_i}^{t_{i+1}}\left( B(t_i) \int _{t_i}^t u(s) {\mathrm{\,d}}s\right) ^{\top } S(t_i) {\mathrm{\,d}}\int _{t_i}^t u(s) {\mathrm{\,d}}s. \end{aligned}$$

Integrating by parts we obtain the relation

$$\begin{aligned}&\int _{t_i}^{t_{i+1}} u(t)^\top \left( B(t_i)^\top S(t_i) + S(t_i)^\top B(t_i)\right) \int _{t_i}^t u(s) {\mathrm{\,d}}s {\mathrm{\,d}}t \\&\quad = \int _{t_i}^{t_{i+1}} u(s)^\top {\mathrm{\,d}}s \, B(t_i)^\top S(t_i) \int _{t_i}^{t_{i+1}} u(s) {\mathrm{\,d}}s. \end{aligned}$$

Following [28], in order to obtain a second-order expansion expressed in term of the first and second-order momentum of *u*, we assume the following.

#### Assumption (I)

The matrix $B^{\top }(t)S(t)$ is symmetric for all $t\in [0,T]$.

Indeed, using Assumption (I) we obtain from the last exposed equality the expression

$$\begin{aligned} \int _{t_i}^{t_{i+1}}\left( B(t_i) \int _{t_i}^t u(s) {\mathrm{\,d}}s \right) ^{\top } S(t_i) u(t) {\mathrm{\,d}}t = \frac{1}{2} \int _{t_i}^{t_{i+1}} u(s)^\top {\mathrm{\,d}}s \, B(t_i)^\top S(t_i) \int _{t_i}^{t_{i+1}} u(s) {\mathrm{\,d}}s, \end{aligned}$$

which can be substituted in (3.5).

Notice that Assumption (I) is always fulfilled if $m=1$. The above obtained second order approximations will be used in the next subsection to define an appropriate discrete-time approximation of problem (1.1)–(1.3).

### The numerical scheme

First of all, observe that the representation (3.1) of *x*(*t*) for $t \in [t_i,t_{i+1}]$ depends on the control *u* only through the integrals $\int _{t_i}^{t_{i+1}} u(t) {\mathrm{\,d}}t$ and $\int _{t_i}^{t_{i+1}} (t-t_i) u(t) {\mathrm{\,d}}t$. The same applies to the approximations of the integral terms of the objective functional obtained in the last subsection. By changing the variable $t = t_i + h s$, this pair of integrals can be represented in the form $h z_1$ and $h^2 z_2$, respectively, where

$$\begin{aligned} z_1= \int _0^1 \varphi (s) {\mathrm{\,d}}s, \quad z_2= \int _0^1 s \varphi (s) {\mathrm{\,d}}s, \end{aligned}$$

and $\varphi (s) = u(t_i+hs)$ is a measurable function with values in $[-1,1]$. By varying *u*, hence $\varphi$, in the set of all admissible controls on [0, *T*], the couple $(z_1,z_2) \in {\mathbb {R}}^{2m}$ generates a strictly-convex and compact set $Z^m\subset {\mathbb {R}}^{2m}$. Note that $Z^m$ can be expressed as the Cartesian product $\prod _{1}^m Z$, where *Z* is the Aumann integral

3.6 $$\begin{aligned} Z := \int _0^1 \left( \begin{array}{cc} 1 \\ s \end{array} \right) [-1,1] d s. \end{aligned}$$

As pointed out in [23], it is a matter of standard calculation to represent the set *Z* in the more convenient way as

$$\begin{aligned} Z= \left\{ (\alpha ,\beta ): \alpha \in [-1,1], \; \beta \in [ \phi _1(\alpha ),\phi _2(\alpha )] \right\} , \end{aligned}$$

where $$\phi _1(\alpha ):=\frac{1}{4}\left( -1+ 2 \alpha +\alpha ^2\right) $$ and $$\phi _2(\alpha ):=\frac{1}{4}\left( 1+ 2 \alpha -\alpha ^2\right) $$.

Following the hint provided by the representation (3.1), we introduce the notations

$$\begin{aligned} \begin{aligned}&A_i := A(t_i) + \frac{h}{2}(A(t_i)^2 + A'(t_i)), \\&B_i := B(t_i) + h A(t_i) B(t_i), \quad C_i := -A(t_i)B(t_i) + B'(t_i), \end{aligned} \end{aligned}$$

and replace the differential equation (1.2) with the discrete-time controlled dynamics

3.7 $$\begin{aligned} x_{i+1}= & {} x_i + h (A_i x_i +B_i u_i + h C_i v_i ), \quad i=0,\ldots ,N-1, \quad x_0 \text{ given } ,\qquad \end{aligned}$$

3.8 $$\begin{aligned}&(u_i ,v_i )\in Z^m \quad i=0,\ldots ,N-1. \end{aligned}$$

Taking into account the approximations of the objective functional in the previous subsection, we introduce its discrete-time counterpart: for $x=(x_0,\ldots ,x_N)$, $u=(u_0,\ldots ,u_{N-1})$, $v=(v_0,\ldots ,v_{N-1})$,

3.9 $$\begin{aligned} J^h(x,u,v):= & {} \frac{1}{2} x_N^{\top }( Q x_N +q) \nonumber \\&+\,\frac{h}{2} \sum _{i=0}^{N-1} \left( x_i^{\top }W(t_i) \left( x_i+ h A(t_i)x_i \right) + \frac{h}{2} x_i^{\top }W'(t_i)x_i \right) \nonumber \\&+\, h \sum _{i=0}^{N-1} \left( h x_i^{\top }W(t_i) B(t_i) ( u_i - v_i ) + x_i^{\top } \left( S(t_i) u_i + h S'(t_i) v_i \right) \right. \nonumber \\&\left. +\, h ( A(t_i)x_i )^{\top } S(t_i) v_i \right) +\frac{h^2}{2} \sum _{i=0}^{N-1} \langle B(t_i)^\top S(t_i) u_i, u_i \rangle . \end{aligned}$$

We denote by ($P^h$) the discrete problem of minimizing (3.9) subject to (3.7)–(3.8). The Karush–Kuhn–Tucker theorem gives the following necessary conditions for optimality of $(x_0, \ldots , x_N)$, $(w_0,\ldots , w_{N-1})$, with $w_i := (u_i, v_i) \in Z^m$: there is an (adjoint) sequence $(p_0,\ldots , p_N)$ such that

3.10 $$\begin{aligned}&0 = -x_{i+1}+x_i + h (A_i x_i +B_i u_i + h C_i v_i ), \end{aligned}$$

3.11 $$\begin{aligned}&0 = -p_i + \left( I + h A_i^{\top }\right) p_{i+1} + h \left( S(t_i)u_i + h S'(t_i)v_i+ h A(t_i)^{\top }S(t_i)v_i \right) \nonumber \\&\qquad + \,h \left( W(t_i) + \frac{h}{2} W(t_i) A(t_i)+ \frac{h}{2} A(t_i)^{\top }W(t_i) + \frac{h}{2} W'(t_i)\right) x_i \nonumber \\&\qquad + \,h^2 W(t_i)B(t_i)( u_i - v_i ), \end{aligned}$$

3.12 $$\begin{aligned}&0 \in N_{Z^m}(w) \nonumber \\&\qquad + \left( \begin{array}{c} B^{\top }_i p_{i+1} + S(t_i)^{\top }x_i+ h B(t_i)^{\top }W(t_i)x_i + h B(t_i)^{\top } S(t_i) u_i\\ h \left( C_i^{\top }p_{i+1} - B(t_i)^{\top } W(t_i)x_i + \left( S(t_i)^{\top }A(t_i) +S'(t_i)^\top \right) x_i \right) \\ \end{array} \right) , \end{aligned}$$

3.13 $$\begin{aligned}&0 = -p_N+ Q^{\top }x_N+q. \end{aligned}$$

In order to obtain (3.12) we use again Assumption (I).

### Construction of continuous-time controls and order of convergence

Let $\{(x_i,u_i,v_i,p_i)\}$ be any solution of system (3.10)–(3.13). Based on the sequence $\{(u_i,v_i)\}_{i=0}^{N-1}$ we shall construct a continuous-time admissible control *u* such that

3.14 $$\begin{aligned} \int _{t_i}^{t_{i+1}} u(s) d s = h u_i, \quad \int _{t_i}^{t_{i+1}} (s-t_i) u (s) d s = h^2 v_i, \quad i=0, \ldots , N-1. \end{aligned}$$

The construction is by idea similar to that in [23] with the essential difference that now *u* takes values only in the set $\{-1,1\}$ and the construction is simpler.

For $(\alpha , \beta ) \in Z$, with $\alpha \not = -1$ (that is, $\alpha \in (-1, 1]$) and $\beta \in [\phi _1(\alpha ), \phi _2(\alpha )]$ define

$$\begin{aligned} \tau (\alpha ,\beta ) := \frac{1+2\beta }{2(1+\alpha )} - \frac{1+\alpha }{4}, \qquad \theta (\alpha ,\beta ) := \frac{1+2\beta }{2(1+\alpha )} + \frac{1+\alpha }{4}. \end{aligned}$$

For $\alpha = -1$ we set $\tau = \theta =0$. (Given that $\beta \in [\phi _1(\alpha ), \phi _2(\alpha )]$, this is, in fact, an extension by continuity.) Clearly, $\tau \le \theta$, while $\tau \ge 0$ is implied by $\beta \ge \phi _1(\alpha )$ and $\theta \le 1$ is implied by $\beta \le \phi _2(\alpha )$. Then define the admissible control *u* component-wise as follows: for $j = 1, \ldots , m$ and $i = 0, \ldots , N-1$ set $\tau _i^j = \tau (u^j_i,v^j_i)$, $\theta _i^j = \theta (u^j_i,v^j_i)$, and

3.15 $$\begin{aligned} u^{j}(t) := \left\{ \begin{array}{ll} -1 &{}\quad \text{ if } t \in [t_i, t_i + h \tau _i^j), \\ \, \;\;1 &{}\quad \text{ if } t \in [t_i + h \tau ^j_i, t_i + h \theta ^j_i],\\ -1 &{} \quad \text{ if } t \in (t_i + h \theta ^j_i, t_{i+1}]. \end{array} \right. \end{aligned}$$

The functions $\tau (\cdot )$ and $\theta (\cdot )$ are defined in such a way that the relations (3.14) are fulfilled. To show this, it is enough to substitute the above defined $u(\cdot )$ in (3.14) and calculate the integrals. We skip this trivial but cumbersome calculation.

We mention that in our framework the pairs $(u_i^j, v_i^j)$, $i=0, \ldots , N-1$, $j = 1, \ldots , M$, typically belong to the boundary of the set *Z*. In such a case every component of the control *u* defined in (3.15) has at most one switching point per mesh interval $[t_i,t_{i+1}]$ and we can distinguish the following possibilities:

- if $u_i^j = -1$ or $u_i^j = 1$, then $u^j(t) = -1$, respectively $u^j(t) = 1$, in $[t_i,t_{i+1}]$;
- if $u_i^j \in (-1,1)$ and $v_i^j = \phi _1(u_i^j)$ then $\tau _i^j = 0$, $\theta _i^j = (1+u_i^j)/2$, thus
  3.16 $$\begin{aligned} u^{j}(t) := \left\{ \begin{array}{ll} \, \;\;1 &{}\quad \text{ if } t \in [t_i, t_i + h \theta ^j_i],\\ -1 &{} \quad \text{ if } t \in (t_i + h \theta ^j_i, t_{i+1}]; \end{array} \right. \end{aligned}$$
- if $u_i^j \in (-1,1)$ and $v_i^j = \phi _2(u_i^j)$ then $\tau _i^j = (1-u_i^j)/2$, $\theta _i^j = 1$, thus
  $$\begin{aligned} u^{j}(t) := \left\{ \begin{array}{ll} -1 &{}\quad \text{ if } t \in [t_i, t_i + h \tau ^j_i],\\ \,\;\;1 &{} \quad \text{ if } t \in (t_i + h \tau ^j_i, t_{i+1}]. \end{array} \right. \end{aligned}$$
  A third possibility is that $(u_i^j, v_i^j)$ happens to belong to the interior of *Z*:
- if $u_i^j \in (-1,1)$ and $v_i^j \in ( \phi _1(u_i^j), \, \phi _2(u_i^j) )$ then formula (3.15) has to be used to define $u(\cdot )$.

In fact, formula (3.15) gives a unified description of all the above cases, where some of the three subintervals intervals in (3.15) degenerate in the (typical) cases (i) and (ii).

#### Theorem 3.1

Let Assumption (A1), (A2) and (I) be fulfilled. Let $(\hat{x}, \hat{u})$ be a solution of problem (1.1)–(1.3) for which Assumption (B) is fulfilled with some $\kappa \ge 1$. Let $\hat{p}$ be the corresponding adjoint function (so that $(\hat{x}, \hat{p}, \hat{u})$ satisfies the Pontryagin system (2.1)–(2.4)). Then for every natural number *N* system (3.10)–(3.13) has a solution $\{(x_i,u_i,v_i,p_i)\}$. Moreover, for the continuous embedding of $(u_i,v_i)$ defined in (3.15), it holds that

3.17 $$\begin{aligned} \max _{k=0, \ldots , N} \left( |x_k - \hat{x}(t_k)| + |p_k - \hat{p}(t_k)| \right) + d^{\#} ( u, \hat{u} ) \le c\, h^{2/ \kappa }. \end{aligned}$$

We mention that, for time-invariant problems without singular arcs, Assumption (B) is typically fulfilled with a number $\kappa \in \{1, 2, 3 , \ldots \}$, corresponding to the multiplicity of the zeros of the switching function. As argued in [23], the case $\kappa = 1$ is in a certain sense “generic” and the error estimate (3.17) is of second order in this case. Also in the case $\kappa > 1$ the order of accuracy is doubled in comparison with that proved in [26] for the Euler scheme. Utilization of higher order Runge–Kutta schemes on a fixed mesh could not improve the accuracy of the Euler scheme due to the discontinuity of the optimal control.

A solution $\{(x_i,u_i,v_i,p_i)\}$ of system (3.10)–(3.13) can be obtained by any method for solving the discrete problem (3.7)–(3.9). The adjoint variables $p_i$ do not need to be directly involved. For example, we use for numerical computations a version of the steepest descent method, where the adjoint equation is only indirectly involved for calculation of the derivative of the objective function (3.9) with respect to the variables $(u_i,v_i)$. In any case, the adjoint functions $\hat{p}(\cdot )$ and $\{p_i\}$ are well defined and the error estimate (3.17) is valid.

As we argue in Sect. 5, the solution $\{(x_i,u_i,v_i,p_i)\}$ can be inexact, which leads to a modification of the error estimate as stated there.

## Proof of Theorem 3.1

**1.** Preliminaries.

Let $\{(x_i,u_i,v_i,p_i)\}$ be a solution of the discrete system (3.10)–(3.13) and let $u(\cdot )$ be the continuous embedding of $\{(u_i,v_i)\}$ defined in (3.15).

We embed the sequences $\lbrace x_i \rbrace$ and $\lbrace p_i\rbrace$ into the spaces $W^{1,1}_{x_0}$ and $W^{1,1}$ using the hint provided by the expansions developed in Sect. 3.1. Namely, for $t \in [t_i,t_{i+1})$, we define

4.1 $$\begin{aligned} x(t)&:= \left( I + (t-t_i) A(t_i) + \frac{(t-t_i)^2}{2} (A(t_i)^2 + A'(t_i)) \right) x_i \nonumber \\&\qquad + (B(t_i) + (t-t_i) A(t_i) B(t_i)) \int _{t_i}^t u(s) {\mathrm{\,d}}s + \, C_i \int _{t_i}^t (s-t_i) u(s) {\mathrm{\,d}}s \end{aligned}$$

and

4.2 $$\begin{aligned} p(t)&:= \left[ I + (t_{i+1} -t) A(t_i)^{\top } + \frac{(t_{i+1} - t)^2}{2} A^2(t_i)^{\top } + \frac{h^2-(t-t_i)^2}{2}A'(t_i)^{\top } \right] p_{i+1} \nonumber \\&\qquad +(t_{i+1}-t) W(t_i)x_i +\frac{h^2-(t-t_i)^2}{2} \left( W'(t_i) + W(t_i)A(t_i) \right) x_i \nonumber \\&\qquad +W(t_i)B(t_i) \int _{t}^{t_{i+1}} \int _{t_i}^s u(\tau ) {\mathrm{\,d}}\tau {\mathrm{\,d}}s + \frac{(t_{i+1} - t)^2}{2} A(t_i)^{\top } W(t_i)x_i \nonumber \\&\qquad +A(t_i)^{\top }S(t_i) \int _t^{t_{i+1}} \int _s^{t_{i+1}} u(\zeta ) {\mathrm{\,d}}\zeta \; {\mathrm{\,d}}s + S(t_i) \int _{t}^{t_{i+1}} u(s) {\mathrm{\,d}}s\nonumber \\&\qquad + S'(t_i) \int _{t}^{t_{i+1} } (s- t_i ) u(s) {\mathrm{\,d}}s. \end{aligned}$$

We show below that $p\in W^{1,1}$. From (3.4) and (3.14) it follows that

4.3 $$\begin{aligned} \begin{aligned} \lim _{t\rightarrow t_i+} p(t)&= \left[ I + h A(t_i)^{\top } + \frac{h^2}{2} A^2(t_i)^{\top } + \frac{h^2}{2} A'(t_i)^{\top } \right] p_{i+1} + h W(t_i)x_i\\&\quad \;+\frac{h^2}{2} \left( W'(t_i)x_i + W(t_i)A(t_i)x_i \right) + h^2 W(t_i)B(t_i)(u_i- v_i) \\&\quad \;+ \frac{h^2}{2} A(t_i)^{\top } W(t_i)x_i+ h S(t_i) u_i + h^2 ( A(t_i)^{\top } S(t_i) + S'(t_i)) v_i. \end{aligned} \end{aligned}$$

Since

$$\begin{aligned} \left[ I + h A(t_i)^{\top } + \frac{h^2}{2} A^2(t_i)^{\top } +\frac{h^2}{2} A'(t_i)^{\top } \right] p_{i+1} = \left( I+ h A_i^\top \right) p_{i+1}, \end{aligned}$$

the right-hand side of (4.3) is equal to the expression of $p_i$ given by (3.11). Thus, *p* is continuous at $t_i$, and hence $p\in W^{1,1}$. The proof that $x\in W^{1,1}_{x_0}$ is analogous and can be found in [23, Section 5].

By Theorem 2.2, for every $b > 0$ there exist a number *c* such that if $\Vert y \Vert \le b$ then

4.4 $$\begin{aligned} \Vert x-\hat{x}\Vert _{1,1}+ \Vert p-\hat{p}\Vert _{1,1} + \Vert u-\hat{u}\Vert _1 \le c \Vert y \Vert ^{1/\kappa }, \end{aligned}$$

where $y = (\xi ,\pi ,\nu ,\rho )$ is the residual that (*x*, *p*, *u*) gives in (2.1)–(2.4), that is, $y \in F(x,p,u)$. Thus we have to estimate the norm of this residual.

The estimate $\Vert \xi \Vert _1 \le O(h^2)$ of the first residual is obtained in [23, Section 4], where the primal differential equation is the same as in the present paper. We shall analyze below the residual in the remaining Eqs. (2.2)–(2.4).

**2.** Residual in (2.2) and (2.4).

First, we differentiate the expression in (4.2) for $t\in [t_i,t_{i+1})$:

$$\begin{aligned} \begin{aligned} -\dot{p}(t)&= \left[ A(t_i)^{\top } + (t_{i+ 1} - t) A^2(t_i)^{\top } + (t-t_i) A'(t_i)^{\top } \right] p_{i+1} + W(t_i)x_i\\&\qquad + (t-t_i) \left( W'(t_i)+ W(t_i)A(t_i) \right) x_i + W(t_i)B(t_i) \int _{t_i}^{t} u(s) {\mathrm{\,d}}s\\&\qquad + (t_{i+1}-t) A(t_i)^{\top } W(t_i)x_i + A(t_i)^{\top }S(t_i) \int _t^{t_{i+1}} u(\zeta ) {\mathrm{\,d}}\zeta \\&\qquad +S(t_i) u(t) + S'(t_i) (t- t_i ) u(t)\\&= A(t)^{\top }\left( I +(t_{i+1}-t)A(t_i)^{\top }\right) p_{i+1} \\&\qquad + W(t) x_i + (t-t_i)W(t) A(t_i)x_i +W(t)B(t_i)\int _{t_i}^t u(s) {\mathrm{\,d}}s \\&\qquad +(t_{i+1}-t) A(t)^{\top } W(t_i)x_i + A(t)^{\top }S(t_i) \int _t^{t_{i+1}} u(\zeta ) {\mathrm{\,d}}\zeta \\&\qquad + S(t) u(t)+ O(t;h^2). \end{aligned} \end{aligned}$$

Here and below $O(t;h^2) \le C h^2$ for some constant $C>0$ which is independent of $t \in [0,T]$. Using (4.1) and then (4.2) we obtain that

$$\begin{aligned} -\dot{p}(t)= & {} A(t)^{\top }\left( I +(t_{i+1}-t)A(t_i)^{\top }\right) p_{i+1}+(t_{i+1}-t) A(t_i)^{\top } W(t_i)x_i\\&+\, A(t)^{\top }S(t_i) \int _t^{t_{i+1}} u(\zeta ) {\mathrm{\,d}}\zeta + \, W(t)x(t)+S(t) u(t)+O(t;h^2) \\= & {} A(t)^{\top }p(t) +W(t)x(t)+S(t) u(t)+O(t;h^2). \end{aligned}$$

Hence, we deduce that $\Vert \pi \Vert _{\infty } \le O(h^2)$. Notice that the Eq. (3.12) gives that the residual in (2.4) is zero, that is, $\nu =0$.

**3.** Residual in (2.3).

First of all, we derive a second order expansion of the term $B^{\top }p+S^{\top }x$ appearing in (2.3). By (4.1), (4.2) and the Taylor expansion for *B* and *r* we have

$$\begin{aligned} B^{\top }(t)p(t)+ S(t)^\top x(t)&= \left( B(t_i)+ (t - t_{i})B'(t_i)\right) ^{\top }\Big ( p_{i+1}+(t_{i+1}-t)A(t_i)^{\top } p_{i+1} \\&\quad +(t_{i+1}-t) W(t_i)x_i + S(t_i)\int _t^{t_{i+1}} u(s)\; {\mathrm{\,d}}s \Big ) \\&\quad + \left( S(t_i)+S'(t_i)(t-t_i)\right) ^\top \left( \left( I + (t-t_i)A(t_i) \right) x_i \right. \\&\quad \left. + B(t_i)\int _{t_i}^t u(s)\; {\mathrm{\,d}}s \right) +O(t;h^2). \end{aligned}$$

Then, by using the definition of $B_i$ and $C_i$ we obtain that

$$\begin{aligned} B^{\top }(t)p(t)+ S(t)^\top x(t)&=\left( B_i+ (t-t_i)C_i\right) ^{\top } p_{i+1}+ B(t_i)^{\top }\left( (t_{i+1}-t) W(t_i)x_i\right. \\&\quad \left. + S(t_i)\int _{t}^{t_{i+1}} u(s)\; {\mathrm{\,d}}s \right) + S(t_i)^{\top }B(t_i)\int _{t_i}^t u(s) {\mathrm{\,d}}s\\&\quad + S(t_i)^{\top } \left( I + (t-t_i)A(t_i) \right) x_i \\&\quad + S'(t_i)^{\top }(t-t_i) x_i +O(t;h^2). \end{aligned}$$

Since Assumption (I) means $B^{\top }S=S^{\top }B$,

4.5 $$\begin{aligned} B^{\top }(t)p(t)+ S(t)^\top x(t)&= \left( B_i+ (t-t_i)C_i\right) ^{\top }p_{i+1} \nonumber \\&\quad + B(t_i)^{\top }\left( (t_{i+1}-t) W(t_i)x_i + S(t_i)\int _{t_i}^{t_{i+1}} u(s)\; {\mathrm{\,d}}s \right) \nonumber \\&\quad + S(t_i)^{\top } \left( I + (t-t_i)A(t_i) \right) x_i \nonumber \\&\quad + S'(t_i)^{\top }(t-t_i) x_i +O(t;h^2). \end{aligned}$$

Our goal is now to estimate the norm $\parallel \cdot \parallel _{\infty }$ of the residual in (2.3). Since $N_U (u)=\prod _{j=1,\ldots ,m} N_{[-1,1]}(u^j)$, we analyze a single component *j* of (2.3). Moreover, (2.3) is a point-wise relation, therefore we consider it on an arbitrarily fixed interval $[t_i, t_{i+1}]$. We also mention that the set *Z* is the area surrounded by the two parabolas $\beta = \phi _1(\alpha )$ and $\beta = \phi _2(\alpha )$, where $\phi _1(\alpha ) \le \phi _2(\alpha )$. Thus the normal cone to *Z* is easy to calculate and the following expression is provided in [23, Section 4]:

4.6 $$\begin{aligned} N_Z(\alpha ,\beta ) = \left\{ \begin{array}{cl} \emptyset &{} \quad \text{ if } (\alpha ,\beta ) \not \in Z \\ \{ \alpha ( \lambda ,\mu -\lambda )^\top : \,\mu \ge 0, \lambda \ge 0 \} &{} \quad \text{ if } \alpha \in \{-1, 1\} \\ \{\mu \, (\zeta + \alpha , -2 \zeta )^\top : \,\mu \ge 0 \} &{} \quad \text{ if } \alpha \in (-1,1) \wedge \beta \in \{ \phi _1(\alpha ), \phi _2(\alpha )\} \\ \{ 0 \} &{} \quad \text{ if } \alpha \in (-1,1) \wedge \beta \in (\phi _1(\alpha ), \phi _2(\alpha )), \end{array} \right. \nonumber \\ \end{aligned}$$

where $$\zeta = \mathrm{sgn}(\alpha - 2\beta )$$.

We consider separately each of the cases (i), (ii) and (iii) for construction of continuous time control that appear in Sect. 3.3.

**Case (i)** $u_i^j \in \lbrace -1,1\rbrace$. To be specific, let us assume that $u_i^j = -1$, hence $v_i^j = \phi _1(-1) = \phi _2(-1) = -1/2$. (The case $u_i^j = 1$ is similar). The normal cone to *Z* at the point $(-1, - 1/2)^\top$ is [see the second line in (4.6)]

$$\begin{aligned} N_Z\left( (-1,-1/2)\right) = \left\{ \mu \,(0,-1)^\top + \lambda \, (-1,1)^\top : \,\mu \ge 0, \; \lambda \ge 0 \right\} . \end{aligned}$$

Then due to (3.12) for every $j= 1, \ldots , m$ there exist $\mu \ge 0$ and $\lambda \ge 0$ such that

4.7 $$\begin{aligned}&\left( B^{\top }_i p_{i+1} + S(t_i)^{\top }x_i + h B(t_i)^{\top } W(t_i)x_i + h B^{\top }(t_i) S(t_i) u_i \right) ^j = \lambda ,\end{aligned}$$

4.8 $$\begin{aligned}&h \left( C_i^{\top }p_{i+1} - B(t_i)^{\top }W(t_i)x_i + \left( S(t_i)^{\top }A(t_i) +S'(t_i)^\top \right) x_i \right) ^j = (\mu -\lambda ).\qquad \qquad \end{aligned}$$

Observe that, for $t\in [t_i,t_{i+1}]$,

4.9 $$\begin{aligned} \lambda + (\mu -\lambda ) \frac{(t- t_i)}{h} = \mu \frac{(t-t_i)}{h}+ \lambda \left( 1- \frac{(t-t_i)}{h} \right) , \end{aligned}$$

thus the quantity above is non-negative for all $t\in [t_i,t_i+h)$. Thus, adding up (4.7) and (4.8), the latter multiplied by $(t-t_i)/h$, we obtain that

$$\begin{aligned}&\big [\left( B_i+ (t-t_i)C_i\right) ^{\top }p_{i+1} + B(t_i)^{\top }\left( (t_{i+1}-t) W(t_i)x_i + S(t_i) u_i \right) \\&\quad +\, S(t_i)^\top \left( I + (t-t_i)A(t_i) \right) x_i + S'(t_i)(t-t_i) x_i\big ]^{j} \ge 0. \end{aligned}$$

By (3.14) the quantity above is identical to the *j*-th component of the right-hand side of (4.5), modulo $O(t;h^2)$. By the fact that $u^j(t)=-1$ in case (i), we thus obtain

4.10 $$\begin{aligned} ( B^{\top }p(t)+S(t)^{\top }x(t))^j +O(h^2)\in - N_{[-1,1]} (u^j(t)). \end{aligned}$$

**Case (ii)** $u_i^j \in (-1,1)$, $v_i^j \in \{ \phi _1(u_i^j), \, \phi _2(u_i^j)\}$. We consider the case $v_i^j = \phi _1(u_i^j)$; the case $v_i^j = \phi _2(u_i^j)$ can be treated similarly. The continuous-time control $u^j(\cdot )$ is defined by (3.16), where the jump point $\theta _i^j = (1-u_i^j)/2$. The normal cone to *Z* at $(u_i^j,\phi (u_i^j))$ is (see the third line in (4.6))

$$\begin{aligned} N_Z\left( (u_i^j,\phi (u_i^j))\right) = \left\{ \mu \left( 1+u^j_i,-2\right) ^{\top }: \mu \ge 0 \right\} . \end{aligned}$$

By (3.12), there exists $\mu \ge 0$ such that

4.11 $$\begin{aligned}&\left( B^{\top }_i p_{i+1} +S(t_i)^{\top }x_i+ h B(t_i)^{\top }W(t_i)x_i+ h B(t_i)^{\top } S(t_i)u_i \right) ^j = -\mu (1+ u_i^j), \nonumber \\\end{aligned}$$

4.12 $$\begin{aligned}&h \left( C_i^{\top }p_{i+1}- B(t_i)^{\top } W(t_i)x_i+ \left( S(t_i)^{\top }A(t_i)+S'(t_i)\right) x_i\right) ^j= 2 \mu . \end{aligned}$$

Observe that, from the definition of $\theta ^j_i$, it follows that the quantity

4.13 $$\begin{aligned} -\mu (1+u_i^j ) + 2 \mu \frac{(t-t_i)}{h} \end{aligned}$$

is non-positive whenever $t\in [t_i,t_i+h \theta _i^j)$, and non-negative whenever $t\in [t_i+h \theta _i^j , t_{i+1}]$. Thus, adding up (4.11) and (4.12), the latter multiplied by $(t-t_i)/h$, and using the definition of $u^j$ we obtain that

$$\begin{aligned}&\big ( B^{\top }_i p_{i+1} + S(t_i)^{\top }x_i +h B(t_i)^{\top }W(t_i)x_i + h B(t_i)^{\top }S(t_i)u_i \big )^j \nonumber \\&\quad + (t-t_i)\left( C_i^{\top }p_{i+1} - B(t_i)^{\top } W(t_i)x_i+ \left( S(t_i)^{\top }A(t_i)\right. \right. \\&\quad \left. \left. + S'(t_i)\right) x_i\right) ^j \in - N_{[-1,1]}(u^j(t)). \end{aligned}$$

By (3.14) and (4.5), the left-hand side term of the relation above is equal to $( B^{\top }p(t)+S(t)^{\top }x(t)+r(t))^j +O(h^2)$. This proves (4.10) in the case (ii).

**Case (iii)** By (3.12) and the fact that $(u_k^j,v_k^j) \in \text{ Int } Z$, we have

$$\begin{aligned}&\left( B^{\top }_i p_{i+1} + S(t_i)^{\top }x_i + h B(t_i)^{\top } W(t_i)x_i + h B(t_i)^{\top } S(t_i)u_i \right) ^j =0,\\&h \left( C_i^{\top }p_{i+1}- B(t_i)^{\top } W(t_i)x_i + \left( S(t_i)^{\top }A(t_i) +S'(t_i) \right) x_i \right) ^j=0. \end{aligned}$$

Then,

$$\begin{aligned} 0&=\Big ( B^{\top }_i p_{i+1} + S(t_i)^{\top }x_i + h B(t_i)^{\top } W(t_i)x_i + h B(t_i)^{\top } S(t_i)u_i \\&\quad \left. +\, (t-t_i)\left( C_i^{\top }p_{i+1}- B(t_i)^{\top } W(t_i)x_i + \left( S(t_i)^{\top }A(t_i) +S'(t_i) \right) x_i \right) \right) ^j. \end{aligned}$$

This and (4.5) yield (4.10) in the case (iii). We can finally conclude that $\Vert \rho \Vert _{\infty } = O(h^2)$.

Summarizing, we have obtained that $\Vert y\Vert \le c_1 h^2$, where $c_1$ is independent of *N*. Since $c_1 h^2 \le c_1 T^2 =: b$, Theorem 2.2 implies existence of *c* such that for every natural *N*

$$\begin{aligned} \Vert x - \hat{x} \Vert _{1,1} + \Vert p - \hat{p} \Vert _{1,\infty } + \Vert u - \hat{u} \Vert _1 \, \le \, c \, h ^{2 /\kappa }. \end{aligned}$$

We know that $x(t_i) = x_i$ and $p(t_i) = p_i$, hence

$$\begin{aligned} \max _{i=0, \ldots , N} \left( |x_i - \hat{x}(t_i)| + |p_i - \hat{p}(t_i)| \right) + \Vert u - \hat{u} \Vert _1 \le c_2 \, h^{2/ \kappa }. \end{aligned}$$

Now we focus on the last term in the left-hand side. Since $\hat{u}$ and *u* take only values $\pm 1$, as already pointed out for instance in [25, Section 4], we have that $d^{\#}(u,\hat{u})\le c_3 \Vert u - \hat{u} \Vert _1$ for some $c_3 \ge 0$, and this concludes the proof.

## Error estimate in case of inexact solution of the discrete problem

The estimation (3.17) in Theorem 3.1 is valid on the assumption that the discrete-time problem (3.7)–(3.9) is exactly solved. In the present section we incorporate in the error estimation possible inaccuracy in solving the discrete-time problem. The basic argument for that is identical with the one for Mayer’s problems, presented in [23, Section 5], therefore we only sketch it.

We assume that as a result of a numerical procedure for solving the mathematical programming problem (3.7)–(3.9) we have obtained an approximate solution $(\{\tilde{x}_i \}$, $\{\tilde{p}_i \}$, $\{\tilde{w}_i\})$ of the first order optimality (Karush–Kuhn–Tucker) system (3.10)–(3.13). This means that the relations (3.10)–(3.13) are satisfied by the sequences $(\{\tilde{x}_i \}$, $\{\tilde{p}_i \}$, $\{\tilde{w}_i\})$ with some residual $(\xi , \pi , \rho , \nu ) = \left( \{\xi _i\}_0^{N-1}, \{\pi _i\}_0^{N-1}, \{\rho _i\}_0^{N-1}, \nu \right)$. We measure the size or the residual by the number

$$\begin{aligned} \varepsilon:= & {} \Vert \xi \Vert _{l_1} + \Vert \pi \Vert _{l_\infty } + \Vert \rho \Vert _{l_\infty } + |\nu | = h \sum _{i=0}^{N-1} |\xi _i| \; + \; \max _{i = 0, \ldots , N-1} |\pi _i| \; \\&+ \; \max _{i = 0, \ldots , N-1} |\rho _i| \;+\; |\nu |. \end{aligned}$$

Using the approximate solution $\{\tilde{w}_i\}$, one can define an approximation, $\tilde{u}(\cdot )$, of the optimal control $\hat{u}$ in the same way as described in Sect. 3.3. Then the estimation (3.17) in Theorem 3.1 takes the form

5.1 $$\begin{aligned} \max _{i=0, \ldots , N} \left( |\tilde{x}_i - \hat{x}(t_i)| + |\tilde{p}_i - \hat{p}(t_i)| \right) + d^\#(\tilde{u} - \hat{u}) \le c \,(\varepsilon + h^2)^{ 1/ \kappa }. \end{aligned}$$

The proof of this statement is not straightforward, but the argument is identical with that in [23, Section 5], therefore we do not repeat it here.

Clearly, in order to make the error arising in the proposed discretization scheme and the error in solving the discretized problem consistent, one has to solve the latter with accuracy $\varepsilon$ proportional to $h^2$.

## A numerical experiment

### Example 6.1

Let us consider the following optimal control problem on the plane:

$$\begin{aligned} \min \left\{ -b y(1) + \int _0^1 \frac{1}{2} (x(t))^2 {\mathrm{\,d}}t \right\} \end{aligned}$$

subject to

$$\begin{aligned}&\dot{x} = y, \quad x(0) = a, \\&\dot{y} = u, \quad y(0) = 0, \end{aligned}$$

with control constraint $u \in [-1,1]$ and for $a > 1/2$, $b > 0$.

**Table 1:** Here $e_N = d^{\#}(\hat{u} - \tilde{u}^N)$ is the error of the numerically obtained control $\tilde{u}^N$ for various values of *N*

*N*	10	20	30	40	60
$e_N$	$1.50 \times 10^{-3}$	$3.64 \times 10^{-4}$	$1.54\times 10^{-4}$	$9.35 \times 10^{-5}$	$3.97\times 10^{-5}$
$e_N/h^2$	0.150	0.146	0.139	0.150	0.143

The last line gives the values $e_N/h^2$

Here (for appropriate values of *a* and *b*) there is a unique optimal solution with a switch from $u=-1$ to $u=1$ at time $\tau$ which is a solution of the equation

$$\begin{aligned} -5 \tau ^4 +24 \tau ^3 - (12 a + 36) \tau ^2 + (24 a + 20) \tau + 24 b -12 a -3 = 0. \end{aligned}$$

Moreover, $\tau$ is a simple zero of the switching function, thus $\kappa =1$. Taking, for example, $a = 1$ and $b=0.1$, the equation above becomes

$$\begin{aligned} -5 \tau ^4 + 24 \tau ^3 - 48 \tau ^2 + 44 \tau - 12.6 = 0 \end{aligned}$$

and the single real solution of this equation in [0, 1] is $\tau = 0.492487520$ with all digits being correct. We solved system (3.10)–(3.13) for various values of the discretization step $h = T/N$ (*N* is a natural number) by using a version of the gradient projection method. The computation of the approximate solutions $(\{\tilde{x}_i \}$, $\{\tilde{p}_i \}$, $\{\tilde{w}_i\})$ is done with accuracy (measured by the residual $\varepsilon$—see Sect. 5) higher than $h^2$, so that the theoretical error estimate (5.1) is $c h^2$. As before, $(\hat{x}, \hat{p}, \hat{u})$ is the exact solution of the considered problem, and $\tilde{u}$ is the continuous time control constructed as described in Sect. 3.3 for the computed $\{\tilde{w}_i\} = \{(\tilde{u}_i, \tilde{v}_i)\}$ (note that $\tilde{u}$ depends on size of the mesh *N*, therefore further we use the notation $\tilde{u}^N$ instead of $\tilde{u}$).

In Table 1 we report numerical results, focusing on the most critical error $e_N =d^{\#}(\hat{u} - \tilde{u}^N)$. We also calculate the value $e_N/h^2$, which according to (5.1) should be bounded. This is confirmed by the results.

## Concluding remarks

In this paper we extend the analysis of the discretization scheme introduced in [23] for Mayer’s problems to embrace convex linear-quadratic problems, affine with respect to the control. The optimal controls are assumed to be purely bang–bang, with an additional assumption involving the associated “switching function”. Our discretization approach opts for solving a discrete-time optimization problem involving an additional control variable. This yields an order of convergence which doubles that of the Euler’s method. The price for that is that the discrete problem involves quadratic control constraints (instead of the box-type constraints in the original problem).

It is worth noting that the components of the optimal controls could be, in general, concatenations of bang–bang and singular arcs. This challenging case will be a subject of further investigation. It requires, among other things, a deeper analysis of the metric sub-regularity of the system of first order optimality conditions under perturbations (a step in this direction is made in [13]). Another challenging issue is to avoid Assumption (I). Assumption of this kind is present also in [29], as well as in [16], in a nonlinear context, where it requires the Lie brackets of the involved controlled vector fields to vanish. An idea in this direction is presented in [19], but it does not seem to be efficient for numerical implementation.

## Footnotes

1 To avoid a confusion, we mention that the admissible controls, hence the derivative of the state function, $\dot{x}$, as well as the derivative of the adjoint function, $\dot{p}$, appearing below, belong to $L^\infty$. However, we use the $L^1$-norms of these derivatives in most of the considerations.
